# Light-Induced Actuation of Poly(dimethylsiloxane) Filled with Graphene Oxide Grafted with Poly(2-(trimethylsilyloxy)ethyl Methacrylate)

**DOI:** 10.3390/polym10101059

**Published:** 2018-09-24

**Authors:** Josef Osicka, Miroslav Mrlik, Markéta Ilčíková, Lukas Munster, Pavel Bazant, Zdenko Špitalský, Jaroslav Mosnáček

**Affiliations:** 1 Centre of Polymer Systems, University Institute, Tomas Bata University in Zlin, Trida T. Bati 5678, 760 01 Zlin, Czech Republic; osicka@utb.cz (J.O.); munster@utb.cz (L.M.); bazant@utb.cz (P.B.); 2 Polymer Institute, Slovak Academy of Sciences, Dubravska cesta 9, 845 41 Bratislava, Slovakia; marketa.ilcikova@savba.sk (M.I.); upolspiz@savba.sk (Z.Š.); 3 Centre for Advanced Materials Application, Slovak Academy of Sciences, Dubravska cesta 9, 845 45 Bratislava, Slovakia

**Keywords:** light-induced actuation, SI-ATRP, graphene oxide, reduction, dielectrics, dynamic mechanical analysis

## Abstract

This study serves to combine two approaches into one single step, to achieve a significant improvement of the light-induced actuation capabilities. Graphene oxide (GO) is an inert material, from the electrical and thermal conductivity point of view, and is incompatible with the usually-used poly(dimethylsiloxane) (PDMS) matrix. During surface-modification by surface-initiated atom transfer radical polymerization, the GO was transformed into a conducting and compatible material with the PDMS showing enormous light-induced actuation capability. The GO surface-modification with poly(2-(trimethylsilyloxy)ethyl methacrylate) (PHEMATMS) chains was confirmed by transmission electron microscopy and thermogravimetric analysis, with an on-line monitoring of gasses using FTIR. The improved compatibility was elucidated using contact angle and dielectric properties measurements. The PHEMATMS shell was investigated using gel permeation chromatography and nuclear magnetic resonance. The improved electric conductivity was measured using the four-point probe method and by Raman spectroscopy. The very important mechanical properties were elucidated using dynamic mechanical analysis, and with the help of thermo-mechanic analysis for the light-induced actuation. The excellent actuation capabilities observed, with changes in the length of around 0.8% at 10% pre-strain, are very promising from the point of view of applications.

## 1. Introduction

Materials capable of changing its dimensions, upon encountering certain external stimulus, reversibly belongs to the group of smart materials [1,2]. Such deformation can be controlled using various stimuli such as electric [3,4] or magnetic field [5,6], humidity [7,8], temperature [9,10], and light [11,12,13]. From these stimuli, only the humidity, temperature and light-induced deformation provide a non-contact deformation, thus, creating various very-specific applications [14,15].

Materials that show a light-induced capability usually consist of two phases, the filler, and the polymer matrix. The former includes mainly organic [16,17] and carbon-based fillers [18,19,20], while the latter can be formed by various systems. The most frequent matrix is poly(dimethylsiloxane) elastomer (PDMS) [20], but in some cases, the thermoplastic elastomers [13], polyurethanes [21], or rubber compounds [22] have been successfully applied.

In order to obtain materials with sufficient performance, the stiffness of the matrix is a crucial parameter [20]. There are various methods of providing a material with lower stiffness and improved elongation, such as addition of the low molecular weight liquids [18] into the matrices, optimizing the amount of cross-linking agent, in the case of chemically cross-linked systems [6], or designing the chemical composition of the thermoplastic elastomers by the means of a length of hard and soft segments [13].

In the case of filler, the crucial parameter is the absorption of light with a wavelength of the utilized light source [23], a good compatibility with the matrix ensuring maximal heat transfer to the matrix and proper dispersion, in the matrix, providing an enhanced thermal conductivity, and homogeneous distribution of the heat within the matrix [24].

Several techniques have been proposed to fulfill the mentioned recommendations. These include a physical modification of the filler surface by using surfactants, [25], covalent modification of the filler with low molecular weight compounds or polymeric chains compatible with polymer matrix [26], and in situ grafting of the filler, during the synthesis of the polymer matrix [13].

In our preceding study, we have shown the influence of the structure of the polymer chains—such as, poly(methyl methacrylate) (PMMA), poly(*n*-butyl methacrylate) (PBMA), and poly(glycidyl methacrylate) (PGMA) which are grafted onto the surface of the graphene oxide (GO) particles—on the final actuation capability of the PDMS-based photo-actuators [18,27]. Additionally, the effect of the filler concentration [18] and the polymer chain length [27] has already been investigated.

In this study, in order to further improve the compatibility with the polymer matrix, the poly(2-(trimethylsilyloxy)ethyl methacrylate) (PHEMATMS) was grafted from the surface of GO particles and mixed with PDMS and a certain amount of silicon oil, to improve the flexibility of the matrix. Successful modification of GO surface was confirmed using transmission electron microscopy and thermogravimetric analysis, with on-line monitoring of gasses, using FTIR. The compatibility was elucidated using contact angle investigations and dielectric properties. Mechanical performance and light-induced actuation were investigated by the means of dynamic mechanical analysis and modified thermo-mechanical analysis, respectively. The application of the surface-initiated atom transfer radical polymerization (SI-ATRP) technique for grafting of the PHEMATMS onto the GO surface, can provide partially reduced and polymer-modified GO with an improved compatibility for the PDMS matrix, leading toward an excellent light-induced actuation, especially for filler concentrations as low as 1 vol.%. According to our knowledge, utilizing silane-based grafts on the surface of GO and mix them into the PDMS elastomer, for an improvement of the light-induced deformations, has not been published.

## 2. Materials and Methods

### 2.1. Materials

Graphite (powder, <20 μm, synthetic) as a precursor of GO; sulfuric acid (H_2_SO_4_, reagent grade, 95–98%), sodium nitrate (NaNO_3_, ACS reagent, ≥99%), potassium permanganate (KMnO_4_, 97%) and hydrogen peroxide (H_2_O_2_, ACS reagent, 29–32 wt % H_2_O_2_ basis) were used as chemical agents for the GO sheets formation. GO particles were fabricated by the modified Hummers method from graphite powder, as was described previously [28]. α-Bromoisobutyryl bromide (BiBB, 98%) was as an initiator, linked onto the GO surface and the triethyleneamine (TEA, ≥99%), as a proton trap. The GO sheets were modified with atom transfer radical polymerization (ATRP) initiator as described previously [29]. 2-(trimethylsilyloxy)ethyl methacrylate (HEMATMS, 99%), ethyl α-bromoisobutyrate (EBiB, 98%), *N*,*N*,*N′*,*N″*,*N″*-pentamethyldiethylenetriamine (PMDETA, ≥99%), copper bromide (CuBr, ≥99%), and anisole (99%) were used as a monomer, sacrificial initiator, ligand, catalyst, and solvent, respectively, for the ATRP. Diethyl ether (ACS reagent, anhydrous, ≥99%) was used to dry the semi and the final product. All chemicals were purchased from Sigma Aldrich (St. Louis, MO, USA). For the polymer matrix, polydimethyl siloxane Silgard 184 (PDMS) from Dow Corning (Midland, MI, USA), and dried silicone oil M200 from Lukosiol (Kolin, Czech Republic) were used. Tetrahydrofuran (THF, p.a.) dried by flakes of sodium (99.9%), dimethyl formamide (DMF, p.a.), acetone (p.a.), ethanol (absolute anhydrous, p.a.), toluene (p.a.), and hydrochloric acid (HCl, 35%, p.a.) were obtained from Penta Labs (Prague, Czech Republic). Deionized water (DW) was used in all the experiments.

### 2.2. Surface Initiated Atom Transfer Radical Polymerization

The initiator–modified GO sheets (1 g) were put into a Schlenk flask and evacuated and backfilled with argon, three times. HEMATMS (146.6 mmol, 32 mL), EBiB (1.466 mmol, 0.215 mL), PMDETA (5.864 mmol, 1.22 mL), and anisole (32 mL) were pre-purged with argon, at least for 10 min each, and added into the flask, under an argon flow. The system was degassed by three freeze–pump–thaw cycles and finally filled with argon. The catalyst CuBr (1.466 mmol, 0.2103 g) was added to the frozen system under a gentle argon flow. The molar ratio of the reactants [HEMATMS]:[EBiB]:[CuBr]:[PMDETA] was [100]:[1]:[1]:[4]. Anisole was used as a solvent in an amount of 50 vol %. The flask with the polymerization mixture was immersed in a 60 °C preheated, silicone oil bath, to initiate the polymerization process, and stirred at this temperature for two hours. Finally, the polymerization was stopped by exposing the mixture to air. The product, GO–PHEMATMS, was filtered, cleaned by the DMF (2 × 200 mL) and acetone (2 × 200 mL), then dried by diethyl ether (2 × 100 mL).

### 2.3. Elastometric Matrix Preparation

The polymer matrix was prepared by mixing the PDMS, silicone oil (SO), and curing agent in a volume ratio of 8:2:1. The matrix was filled by GO–PHEMATMS, in concentrations of 0.1 vol %, 0.5 vol %, and 1 vol %, and properly homogenized using a combination of ultra-sonication (UPS-400, Ultra Autosonic, Maharahstra, India) and mechanical stirring with a glass stick. This mixture was poured into a teflon-lined mold and evacuated in order to eliminate the presence of air bubbles. Then the mold was placed in an oven for two hours, at 60 °C, to fully cross-link the PDMS-based samples.

### 2.4. Analyses

**Figure 1:**
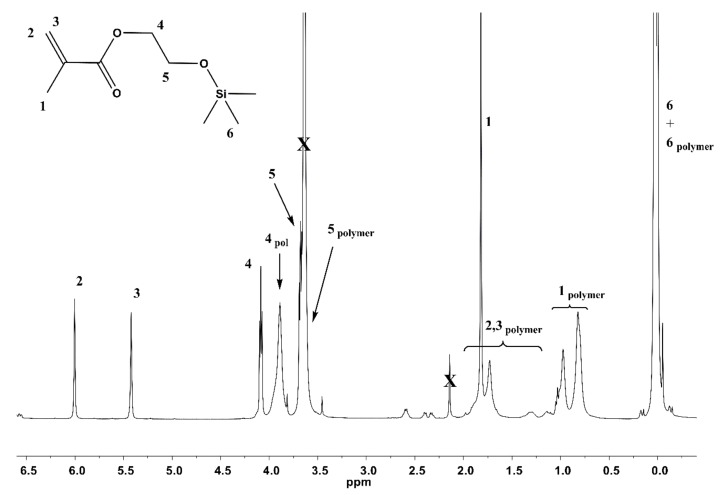
Representative ^1^H NMR spectrum from filtered polymerization mixture of poly(2-(trimethylsilyloxy)ethyl methacrylate) (PHEMATMS).

The modification of the GO with an initiator and the PHEMATMS chains was proved by FTIR (ATR) Nicolet 6700 (Thermo Scientific, Madison, WI, USA), transmission electron microscopy JEOL JEM 2100 (JEOL, Tokyo, Japan), and Raman spectroscopy Nicolet DXR (Thermo Scientific, USA). The molar mass and dispersity (*Đ*) of PHEMATMS chains were investigated using a gel permeation chromatography (GPC) on the GPC instrument (PL-GPC220, Agilent, Hachioji, Japan), equipped with GPC columns (Waters 515 pump, two PPS SDV 5 μm columns (diameter of 8 mm, length of 300 mm, 500 Å + 105 Å)), and a Waters 410 differential refractive index detector tempered to 30 °C. THF dried over KOH was used as a solvent with a polystyrene calibration. The ^1^H NMR was used to determine the monomer conversion from the ratio of an area of the peak at 3.88 ppm, assigned to the PHEMATMS to the sum of an area of the peaks at 3.88 and 4.08 ppm, where the second one was assigned to the HEMATMS (Figure 1). Contact angle measurement (CA) was evaluated from the static sessile drop method carried out on a Surface Energy Evaluation system, equipped with a CCD camera (Advex Instruments, Brno, Czech Republic). The dielectric properties were measured by Broadband Dielectric Impedance Analyzer (Novocontrol, Montabaur, Germany), in the frequency range of 0.01 Hz to 10 MHz, and temperature range of −150 to 100 °C, using a standard sample cell BDCS 140. The viscoelastic properties of both the nanocomposites and pure polymer matrix were studied by a dynamic mechanical analysis (DMA), in a shear mode.

The glass transition process was evaluated through activation energies calculated from an Arrhenius equation (Equation (1)), in order to see the effect of the modification on the relaxation processes, in the PDMS-based composites:

(1) $$f_{\beta }=f_{\infty }\exp\left(\frac{E_{a}}{k_{B}T}\right),$$

where *E*_a_ is the activation energy, *f*_∞_ is the pre-exponential factor, *T* is the thermodynamic temperature, and *k*_B_ is Boltzmann constant.

In order to properly investigate the polymer chains dynamics, the loss permittivity needed to be recalculated to the loss modulus. This recalculation was performed according to Equation (2),

(2) $$\begin{array}{l} M^{*}=\frac{1}{\varepsilon^{*}}\\ M^{\prime }=\frac{\varepsilon^{\prime }}{{\varepsilon^{\prime }}^{2}+{\varepsilon^{\prime \prime }}^{2}}\\ M^{\prime \prime }=\frac{\varepsilon^{\prime \prime }}{{\varepsilon^{\prime }}^{2}+{\varepsilon^{\prime \prime }}^{2}} \end{array}$$

where ε* is the complex permittivity, *ε′* and *ε″* are relative permittivity and loss permittivity, respectively. M*, *M′*, and *M″* are complex, storage, and loss dielectric moduli, respectively.

The light-induced actuation ability of both the matrix and nanocomposite was investigated using a thermal mechanical analysis (TMA, Mettler Toledo, Langacher, Switzerland), using a process similar to that in Reference [19]. Samples in the form of stripes with dimensions of 15 mm in length, 2.5 mm in width, and 0.26 mm in thickness were irradiated using a red LED diode (Luxeon Rebel, Philips, Amsterdam, The Netherlands). Irradiation was applied for 10 s, at 627 nm, with 6, 9, and 12 mW light source intensity, under 10% pre-strain of the samples. Maximum value of the actuation was characterized by a change in the sample length, during an exposure to light, Δ*L* = (*L*_0_ − *L*)/*L*_0_, where *L*_0_ is the length of a non-irradiated sample mounted between the clamps (10 mm), and *L* is the length of the irradiated sample. Actuation describes a material’s ability to undergo reversible shape changes in response to an external light stimulus.

## 3. Results

The presence of an excess of sacrificial initiator, as compared to initiator bonded onto the GO surface, allowed us to follow the polymerization and to determine the molecular characteristics of the polymer, using GPC, and the monomer conversion, using the NMR spectroscopy (Figure 1). Based on the assumption of the simultaneous growing the polymer chains, in bulk, and from the surface [30], it was found that molar mass and dispersity of the PHEMATMS chains grafted onto the GO surface were 12,600 g·mol^−1^ and 1.19, respectively. The monomer conversion after 2 h of polymerization was 67%. A good correlation of the molar mass with the theoretical one (13,750 g·mol^−1^), calculated from the monomer:sacrificial initiator ratio, and the monomer conversion, as well as the narrow dispersity, confirmed a good control of the ATRP process. The polymerization procedure for comparing the effect of the various grafts on the overall physical properties of the PDMS/hybrid-based composites, was similar to that used in our previous studies [18,27], with regards to the targeting of the PHEMATMS molar mass. It was also found in our preceding study [6], that compatibility with the surrounding matrix was not significantly changed when the molar mass of the grafted polymer chains was over 12,000 g·mol^−1^.

**Figure 2:**
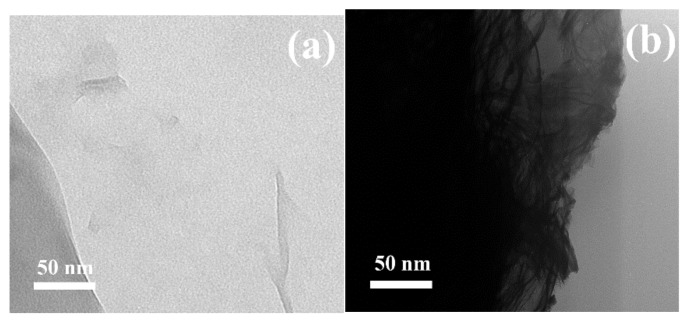
TEM images of (**a**) neat Graphene oxide (GO), (**b**) Graphene oxide grafted with poly(2-(trimethylsilyloxy)ethyl methacrylate) (GO–PHEMATMS).

The successful growth of the polymer chains from the GO surface was confirmed using TEM and TGA, with an online-connected FTIR. As can be seen in Figure 2, the GO was synthesized with considerable exfoliation and only a few layers of GO sheets could be visible in Figure 2a. After the polymerization, the GO particles were analyzed, and certain polymer layers could be observed (Figure 2b) as a floss-like layer with a darker tone than that of a neat GO, and confirmed the presence of polymer chains.

**Figure 3:**
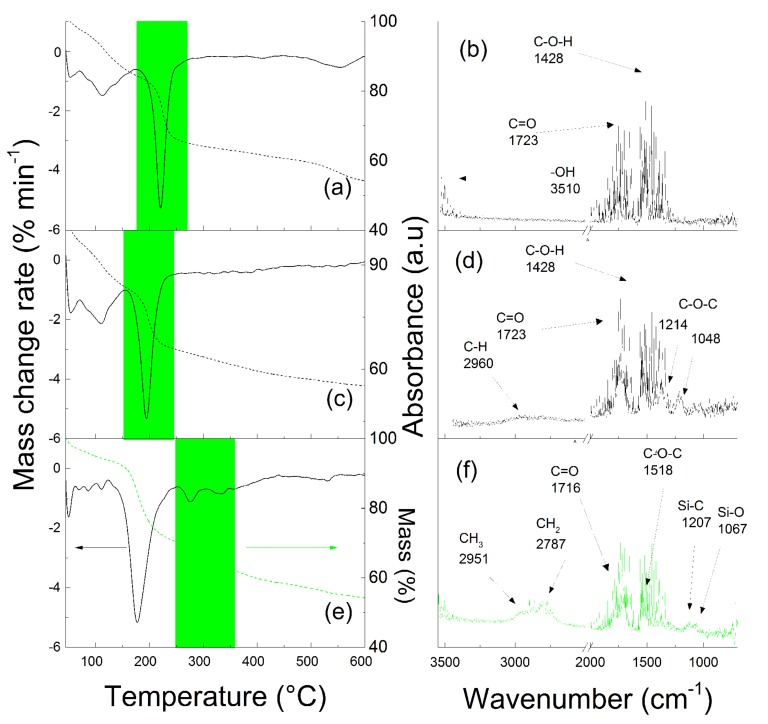
TGA analysis (**a**,**c**,**e**) with on-line monitoring of volatile degradation products from the green marked temperature range by the FTIR spectra (**b**,**d**,**f**) for a neat GO (**a**,**b**), GO-initiator and (**c**,**d**) and GO–PHEMATMS (**e**,**f**) particles.

In order to further confirm the presence of the covalently bonded PHEMATMS shell on the GO surface, the TGA spectra with the FTIR online monitoring of the volatile products produced during the TGA of the GO (Figure 3a,b), initiator-modified GO (Figure 3c,d), and GO–PHEMATMS (Figure 3e,f) particles were analyzed. Properly described decomposition of the neat GO and GO-I has already been published elsewhere [18,27]. In addition to the peak corresponding to the oxygen-containing groups (which was the case for the GO–PHEMATMS shifted to lower temperatures (peak below 200 °C), as compared to the GO and initiator-modified GO), the TGA of the GO–PHEMATMS was characteristic with two more peaks at 270 °C and 330 °C. These peaks corresponded to the volatile products of the thermal degradation of the PHEMATMS chains from the GO surface. The gas phase produced at these critical temperatures range was analyzed using an FTIR (Figure 3f). The FTIR spectra analysis showed the absorption bands typical for methacrylates, as have already been described during the use of this technique [18,27]. Additional peaks at 1207 and 1067 cm^−1^, corresponding to the Si–C and Si–O stretching vibrations, respectively, were also observed and were connected to the trimethylsilyloxyethyl moieties. These results clearly showed the presence of covalently-bonded PHEMATMS on the surface of the GO particles.

**Figure 4:**
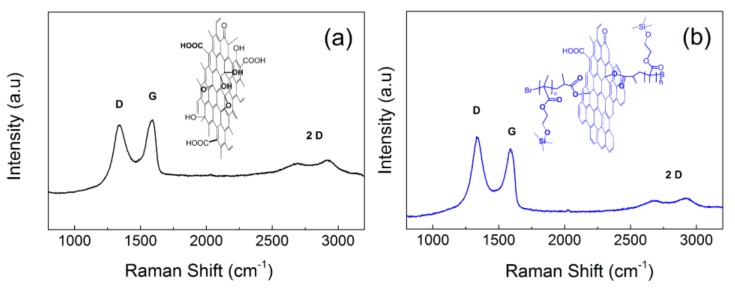
Raman spectra of the neat GO (**a**) and GO-PHEMATMS (**b**) particles and corresponding chemical structures.

In addition to surface modification of the GO, the SI-ATRP approach also provided a simultaneous reduction of the GO surface, as was invented and properly described by Ilcikova et.al. [31]. The degree of reduction could be tailored by the reaction time and the ligand concentration. In this case, the reaction was carried out for two hours and therefore a just negligible GO reduction was obtained. Such a reduction was confirmed by the conductivity measurements, as well as by the change in the *I*_D_/*I*_G_ peaks ratio in the Raman spectra. The peak ratios were 0.9 and 1.09 for the neat GO (Figure 4a) and the GO–PHEMATMS (Figure 4b), respectively, with conductivities of 1.2 × 10^−8^ and 6 × 10^−7^ S·cm^–1^, respectively.

**Figure 5:**
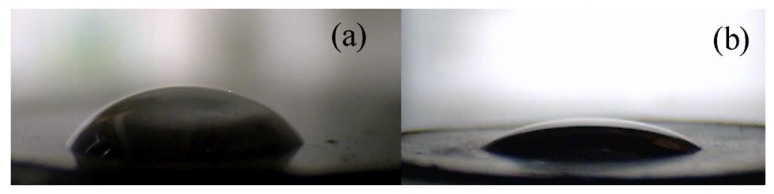
Images from a CCD camera of the 5 µL poly(dimethylsiloxane) (PDMS) droplets on the neat GO (**a**) and the GO-PHEMATMS (**b**).

Compatibility between the filler and the polymer matrix was a crucial parameter to obtain an excellent photo-actuation capability. Therefore, the contact angle investigations were performed between the GO or the GO–PHEMATMS pellet and a drop of the PDMS elastomer. As can be seen in Figure 5a, the neat GO surface showed a contact angle of 49.9° ± 3.2°, indicating only partial wettability and thus only a slight compatibility with the PDMS. On the contrary, the GO–PHEMATMS showed improvement, and in fact, excellent wettability with the PDMS, as proved by the contact angle value of 26.3° ± 3.0°. The wettability observed for the GO–PHEMATMS was also the lowest, in comparison with GO grafted with other types of polymers, where contact angle values of 40.1° ± 1.3° were described for the GO modified with PGMA [27], 38.7° ± 2.7° for PMMA, and 28.7° ± 2.7° for PBMA chains. Such significantly improved compatibility serves a very promising dispersibility of the filler, due to the compatible grafts of the PHEMATMS with the PDMS and can also predict the excellent light-induced material deformation.

**Figure 6:**
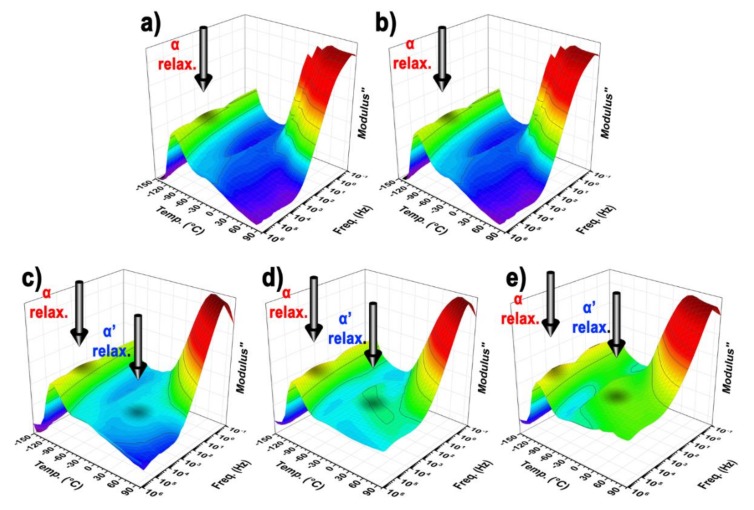
3D plots of the dielectric properties of the neat PDMS matrix (**a**), and PDMS matrix filled with GO (**b**), 0.1 vol % GO–PHEMATMS (**c**), 0.5 vol % GO–PHEMATMS (**d**) and 1 vol % GO–PHEMATMS (**e**).

Dielectric properties are very often used as tools for investigation of polymer chain dynamics. In this case, the polymer chain flexibility was a crucial factor influencing the light-induced deformation. Therefore, the dielectric loss modulus was plotted against the temperature and the frequency, as a 3D plot, to see the differences in the dielectric behavior (Figure 6).

The flexibility of the polymer matrix was given by two factors, the mobility of the main chain characterized by an α relaxation, corresponding to *T*_g_, and the side chains mobility characterized by α′ relaxation, indicating how the entanglements of the side chains got stiff. Therefore, both relaxations were investigated using the Arhenius equation (Equation (1)). The activation energy of α relaxation (Table 1) was significantly shifted to lower values, from 45.57 kJ·mol^−1^ to 22.01 kJ·mol^−1^, after the PHEMATMS grafting, indicating an improved flexibility of the polymer matrix. However, for the side chains flexibility, α′ relaxation, was much more influenced and was clearly visible only for the composites containing the GO–PHEMATMS (Figure 6). Such flexibility was also confirmed by an activation energy decrease from 19.96 kJ·mol^−1^ and 19.09 kJ·mol^−1^ for the neat matrix and composite-containing GO, respectively, to values below 11 kJ mol^−1^, determined for the composites containing the various GO–PHEMATMS loadings (Table 1).

**Figure 7:**
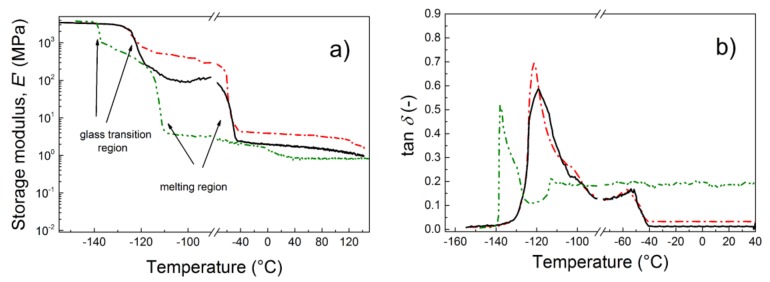
Dependence of the storage modulus (**a**) and tan δ (**b**) for a broad temperature range for a neat PDMS (black solid line), and for PDMS composites containing 0.1 vol % of either the neat GO (red dash–dot line) or the GO–PHEMATMS (green dash–dot–dot line).

The light-induced deformation of the materials was a reversible process and could also be classified as a cyclic deformation process. Therefore, a dynamic mechanical analysis of the neat matrix and various composites was performed. From the mechanical point of view, the GO–PHEMATMS composites showed the lowest values of elastic modulus, due to the significantly increased flexibility of the main, as well as the side polymer chains (Figure 7a). This could also be clearly seen from Figure 7b, where the peak of the *T*_g_ had shifted 15 °C toward lower temperatures, than those of the neat polymer matrix and the nanocomposite-containing unmodified GO. Additionally, the crystalline phase was found to have shifted significantly, with a melting point at a lower temperature. On the other hand, the composite containing GO–PHEMATMS exhibited excellent damping performance with tan delta values of around 0.2, which was classified as a good material for damping applications [6].

**Figure 8:**
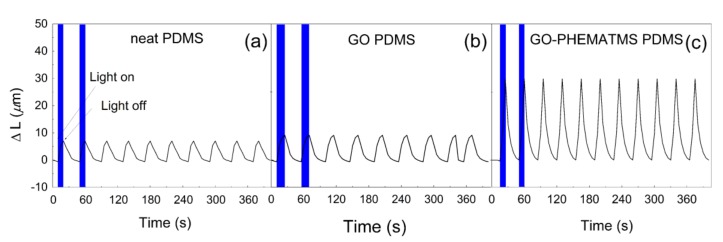
Light-induced deformation capability of pure PDMS (**a**), PDMS composites containing 0.1 vol % of GO (**b**), and GO–PHEMATMS (**c**) under the application of irradiation, with an intensity of 6 mW.

**Figure 9:**
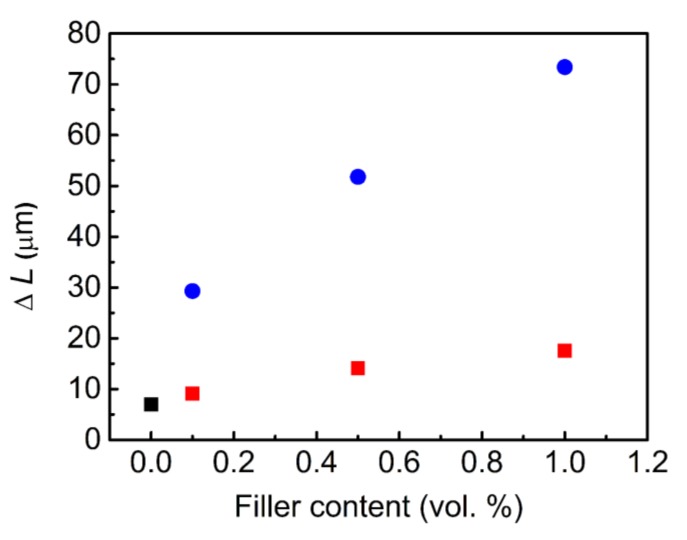
Dependence of the filler content on the change in length, for pure PDMS matrix (black squares), PDMS composites containing unmodified GO (red squares), and GO-PHEMATMS (blue circles). Error bars are within the size of the symbols.

The above-mentioned investigations indicated the significantly enhanced flexibility of the polymer composite due to the grafting of the GO surface with the PHEMATMS chains, and thus, the excellent compatibility with the PDMS. The light-induced deformation studies clearly confirmed these results. As can be seen in Figure 8, the neat PDMS matrix, as well as the composite containing the unmodified GO, showed very poor actuation ability. On the other hand, the composite containing the same vol % of the GO–PHEMATMS exhibited a four times higher actuation-ability, reaching a Δ*L* of 34 μm. For comparison, this value was significantly higher than that recently determined for the composites containing the same vol % of GO–PMMA, GO–PBMA, and GO–PGMA, with Δ*L* values of 9.4, 11.8, and 20.2 μm, respectively [18,27]. In addition, if the concentration of the GO–PHEMATMS filler increased, the ability of the actuation was also significantly improved (Figure 9), reaching values of 55 μm and 75 μm, for 0.5 vol % and 1 vol % of GO-PHEMATMS, respectively. That is, more than six times higher actuation values than those obtained for the composites containing unmodified GO. This superior result was mainly caused by the enormously improved compatibility of the GO–PHEMATMS with the matrix, the proper filler dispersion within the matrix, and the improved flexibility of the matrix in the presence of GO–PHEMATMS.

In these studies, the PDMS was partially softened using an additional amount of SO. However, in order to increase the elasticity and softening of the PDMS matrix, the decreased ratio between the matrix and the curing agent could also be used, as already mentioned in the introduction. Furthermore, the actuation capability was strongly dependent on the matrix stiffness, therefore, the utilization of the hydrogel matrix could also be very effective [32], and will be planned for our future studies, in order to further improve the light-induced actuation of the polymeric materials.

## 4. Conclusions

Surface initiated atom transfer radical polymerization was used for the surface modification of GO nanoparticles with poly(2-(trimethylsilyloxy)ethyl methacrylate) (PHEMATMS) chains, with a molar mass of approximately 12,500 g·mol^−1^. The surface modification was confirmed by TEM and TGA-FTIR. Partial reduction of the GO surface during the polymerization process led to the slight increase of the conductivity from *I*_D_/*I*_G_ 1.2 × 10^−8^ to 6 × 10^−7^ S·cm^–1^, and change of the *I*_D_/*I*_G_ peaks ratio in Raman spectra, from 0.9 for the neat GO to 1.09 for the GO–PHEMATMS. Modification of the GO surface provided a significant improvement in the wettability of the GO surface by the PDMS matrix, as was proved by the contact angle value, which was as low as 26°. This improved compatibility, also dramatically affected the mobility of both the main chains and the side chains. This could be observed by the dielectric measurements which showed a substantial decrease in the activation energies of both α and α′ relaxation processes, which dropped by half, as compared to a pure matrix or composites containing unmodified GO. This could be explained by the effective plasticizing effect of the PHEMATMS chains on the PDMS matrix, thanks to their high wettability. Thus, mechanical properties of the composite containing GO–PHEMATMS were also markedly affected by the increased mobility of the PDMS chains, leading to the decrease of both *T*_g_ and the melting point, by approximately 15 °C and 50 °C, respectively. On the contrary, the damping factor substantially increased after the modification of the GO surface by the PHEMATMS chains. All these results also positively affected the targeted property of the investigated composites, i.e., their actuation performance. The change in the length of the sample, during the light-induced actuation, was found to be four times higher for the composites containing 0.1 vol %, of the GO–PHEMATMS, as compared to the pure PMDS or composites containing the same content of the unmodified GO. Moreover, the actuation ability of the composites containing the GO–PHEMATMS was also significantly enhanced, as compared to all previously published composites, containing GO modified with various polymer chains. Such superior actuation performance is very promising for the application of these materials in various smart systems, sensors, and can be generally utilized in many processes where the non-contact stimulation is a benefit.

## Figures and Tables

**Table 1:** Activation energies of glass transition process for pure PDMS and various PDMS composites. Filler content is in vol %.

Sample Name	Ea α′ (kJ·mol^−1^)	Ea α (kJ·mol^−1^)
PDMS	19.96	45.70
PDMS/GO 0.1%	19.04	36.57
PDMS/GO-PHEMATMS 0.1%	10.7	22.01
PDMS/GO-PHEMATMS 0.5%	9.23	21.3
PDMS/GO-PHEMATMS 1%	9.09	20.1
